# Enhancing Lesion Detection With High Field MRI, How Much Can We?

**DOI:** 10.1177/15357597261466898

**Published:** 2026-07-27

**Authors:** Eliane Kobayashi

**Affiliations:** Department of Neurology, Peter O'Donnell Jr. Brain Institute, University of Texas Southwestern Medical Center

## Abstract

**Clinical 7T MRI for Epilepsy: A Retrospective Review of 50 Cases**

Brian J Burkett, Lily C Wong-Kisiel, Mayur Chalia, Ananya Panda, Karl N Krecke, Steven A Messina, Robert J Witte, Jeffrey W Britton, Benjamin H Brinkmann, Andrew J Fagan, Jason T Little, Theodore J Passe, Robert E Watson, Kirk M Welker. *American Journal of Neuroradiology*. 2026 Jan;47(1):252-261. doi: 10.3174/ajnr.A8924

Background and purpose: Seven Tesla magnetic resonance imaging (7 T MRI) has specific technical features that are advantageous for epilepsy. This study aims to evaluate whether new potentially epileptogenic abnormalities can be identified on 7 T MRI in patients with epilepsy with negative 3 T MRI findings.

Materials and methods: Clinical 7 T epilepsy MRI examinations in patients with prior negative 3 T imaging findings were retrospectively reviewed by 3 neuroradiologists. Their consensus 7 T scan interpretations were reviewed by a neurologist for concordance with EEG findings. Descriptive characteristics of any 7 T MRI abnormalities and their locations were recorded. The clinical and EEG findings in subjects with abnormal 7 T scan findings were compared with those of subjects without a 7 T abnormality.

Results: In patients with epilepsy with nonlesional 3 T MRI, new abnormal findings were identified on 7 T MRI in 36% (18/50) of cases. Of the 14 pediatric cases, there were 7 T MRI abnormal findings in 6/14 (42.9%). Across all cases, a total of 21 discrete abnormal findings were identified, including meningoencephaloceles (5/21, 23.8%), cavernous malformations/possible vascular lesions (5/21, 23.8%), focal cortical dysplasia (3/21, 14.3%), gray matter heterotopia (3/21, 14.3%), mesial temporal sclerosis (3/21, 14.3%), 1 indeterminant hippocampal morphology finding (1/21), and 1 case of a diffuse migrational abnormality (1/21). Noninvasive EEG monitoring unit data were concordant with the location of 7 T abnormalities in 60.0% (9/15) of cases with these clinical data available. Participants with generalized seizures (OR, 0.2; 95% CI, 0.041-0.75) and those with multiple seizure types (OR, 0.14; 95% CI, 0.027-0.52) were significantly less likely to have new potentially epileptogenic lesions detected at 7 T. Two 7 T cases with abnormal findings underwent surgical resection with good clinical outcomes (Engel Class IA).

Conclusions: In clinical practice, 7 T MRI revealed additional epileptogenic lesions in 36% of nonlesional 3 T MRI cases.

## Commentary

Advances in diagnostic instrumentation bring hope for better specificity and sensitivity in determining etiology of neurological diseases. In epilepsy, this can be crucial for the nearly 30% of patients with pharmacoresistant seizures being investigated for possible surgical therapy options.

Magnetic resonance imaging (MRI) scanners are now available for clinical use at a 7 Tesla (7 T) magnetic field strength. Compared to previous generation scanners at 3 T, this comes with improved signal-to-noise ratio (SNR), which increases proportionally to the magnetic field strength. Whereas certain MRI sequences benefit from a higher field, others might in fact be negatively impacted.^1^ Thus, clinical hypotheses on the nature and location of the sought abnormality should be kept in mind to most efficiently capitalize on the technology.

In this study, experienced neuroradiologists in higher field MRIs retrospectively qualitatively reviewed clinical 7 T MRI scans from epilepsy patients who had a previously reported negative 3 T MRI. They aimed to determine whether new potentially epileptogenic abnormalities could be identified on a higher field 7 T MRI that might have been missed in the 3 T scans.

All 7 T MRIs (*N* = 50, aged 9.4-65.6 years, 14 pediatric patients) were acquired in a single scanner and included a T1-weighted MPRAGE, a coronal T2-weighted acquisition through the hippocampal formations, coronal T2 FLAIR, double inversion recovery sampling perfection with application optimized contrast, axial T2 FLAIR with fat saturation, an axial T2-weighted acquisition, axial SWI, and axial DWI. The 3 T MRIs were acquired on various MRI scanners with heterogeneous protocols.

The stepwise approach started with a blind review of the 7 T scans. There was no independent blinded review of the 3 T scans at this stage. The 2 neuroradiologists agreed on the 7 T rating in 39/50 scans. The third neuroradiologist reviewed to adjudicate in 11/50 scans (normal vs abnormal) and additional 4/50 scans for the location of abnormalities. Subsequently, they re-reviewed the 7 T scans with limited clinical information (age and seizure semiology) and determined if the identified 7 T abnormality was more visible in 7 T or 3 T by comparing both scans.

After consensus, they labeled the abnormalities as to their location and type of lesion. This review identified abnormalities in 18/50 (36%) of 7 T MRIs. It is likely that if the same team would have reported these 3 T MRIs in the first place, some of these lesions would have also been seen at 3 T, but this remains speculative.

The fact that retrospectively one can now “see” the lesion in a 3 T MRI once a 7 T scan has shown the way, can further enhance the expertise of neuroradiologists. This was however not compared to other imaging and neurophysiology modalities that can also point to the culprit areas, such as positron emission tomography and magnetic source imaging.

It would be in the best interest of our patients, and of healthcare resource utilization, to undergo one and only optimal MRI scanning session, which would bring the highest chances of depicting brain abnormalities and further guide management decisions. Most often, however, epilepsy patients undergo a series of scans, including a standard brain MRI often ordered by the general neurologist, followed by a (hopefully) dedicated epilepsy protocol MRI once evaluated by an epileptologist. Whereas 3 T MRI has been clinically available for longer, some patients might still have access limited to 1.5 T scanners.

A reasonably sized space-occupying lesion will rarely go unnoticed regardless of the MRI field strength and imaging protocol used, but anatomical abnormalities that most often underlie pharmacoresistant seizures are difficult to depict depending on their location, patient's ability to stay still and result in artifact-free images and the acquisition of MRI sequences that optimize visualization of lesions of different natures.

Within the 18 7 T MRIs concluded with abnormal findings in the present study, a total of 21 abnormalities were detected. As expected, focal cortical dysplasia (3/21, 14.3%) lesions were unraveled with the higher imaging resolution. The 7 T MRIs also revealed previously missed findings such as meningoencephaloceles (5/21, 23.8%), cavernous malformations or other vascular lesions (5/21, 23.8%), and diffuse cortical abnormalities (1/21, 4.8%). It would be perhaps expected that focal cortical dysplasia would represent the highest proportion of lesion type on negative 3 T MRI patients in a tertiary center evaluated with 7 T MRI. However, it might be necessary to deploy MRI postprocessing to further maximize lesion detection even in higher field MRIs.^2^

Higher sensitivity hopefully comes along with higher specificity, and in focal epilepsy concordance with electroclinical data validates epileptogenicity of MRI lesions. Fifteen patients with the newly identified 7 T MRI lesion underwent Phase 1 evaluations, but only 9 (60%) of these lesions showed concordance with video EEG data. Five patients also underwent intracranial EEG investigations, of whom only 2 (40%) showed concordance with the 7 T MRI lesion. Two patients achieved seizure freedom after surgical resection of the identified lesion (meningoencephalocele and mesial temporal sclerosis).

Altogether, the study shows a comparable performance in terms of enhanced lesion detection to previous reports in the literature.^3^ The fact that many unraveled lesions failed to show concordance with electroclinical data including intracranial EEG, and that surgical decisions were not frequently pursued, suggests that higher field MRI confers more sensitivity but could also identify incidental findings, clinical correlation remaining crucial.

It is well justified to deploy more advanced imaging options when other modalities have exhausted identification of a lesion, in particular when 7 T MRI has inherent advantages due to its higher resolution and SNR. Depending on the pulse sequences selected, spatial resolutions as small as 0.25 mm can be achieved, and visualization of vascular structures is enhanced.^1,4^

A 7 T MRI examination might not be the preferred choice for investigation and might not be feasible for all patients, however. One important aspect is patients’ capacity to undergo the examination in a higher field. Motion will impact the images even more so than in a 3 T scanner, thus cooperation to remain still for the duration of the protocol will impact how advantageous (or not) a 7 T MRI will be for each given patient. Another important aspect is the symptom of vertigo, which could limit the patients who would be able to tolerate this higher field imaging scan. Vertigo usually subsides within minutes of walking out of the scanner room, but warrants careful mobilization within the MRI suite to prevent accidents, including falls.^1^
